# Engineering Strategies for Enhanced Extracellular Vesicle Production: Mechanisms and Applications

**DOI:** 10.3390/bios16100555

**Published:** 2026-10-02

**Authors:** Xianyue Ji, Yi Luo, Haozhang Ma, Xingyu Tao, Shuangshuang Wan

**Affiliations:** State Key Laboratory of Flexible Electronics (LoFE), Institute of Advanced Materials (IAM), Nanjing University of Posts & Telecommunications, Nanjing 210023, China

**Keywords:** extracellular vesicles, physical strategies, biochemical strategies, biosensing platforms

## Abstract

Extracellular vesicles (EVs) have emerged as key mediators of intercellular communication and hold great promise for liquid biopsy and therapeutic applications. Their molecular composition and abundance dynamically reflect the pathophysiological state of their parental cells, endowing EVs with significant value for biomarker discovery and precision medicine. However, inefficient biogenesis, limited production yield, and considerable heterogeneity remain core bottlenecks to their clinical translation. This review systematically surveys recent engineering strategies aimed at enhancing EV production, encompassing diverse physical stimuli and biochemical interventions, with a particular focus on the underlying molecular mechanisms that govern EV quantity, composition, and functionality. Despite their varied modes of action, these approaches share a convergent regulatory principle—remodeling the fate of multivesicular bodies (MVBs) by promoting MVB biogenesis, intracellular trafficking, and plasma membrane fusion, while suppressing their diversion toward lysosomal degradation. Importantly, yield-enhancing strategies not only facilitate the scalable manufacturing of therapeutic EVs, but also enable the functional activation of EV-producing cells, enriching disease-relevant molecular signatures and directing the cargo composition for improved diagnostic performance. Crucially, the synergy between yield-enhancing strategies and advanced biosensing platforms is reshaping the entire EV research workflow. Emerging integrated analytical systems—encompassing microfluidics, electrochemical sensors, and acoustic detectors—now enable real-time monitoring of EV secretion kinetics, providing dynamic feedback to optimize stimulation parameters and harvest windows. Moreover, lab-on-a-chip platforms are beginning to merge EV production, in-line purification, and multiplexed detection into a single automated workflow, facilitating simultaneous quality control and functional characterization. Highly sensitive biosensing technologies further allow precise assessment of EV cargo composition, surface marker profiles, and biological activity, ensuring product consistency and potency. Looking forward, the deep integration of EV biogenesis engineering with these smart detection platforms holds the potential to overcome current translational bottlenecks, accelerating the development of scalable EV therapeutics and next-generation liquid biopsy systems for early diagnosis, dynamic disease monitoring, and precision medicine.

## 1. Introduction

Extracellular vesicles (EVs) are a heterogeneous population of membrane-enclosed particles released by virtually all cell types, with sizes spanning from ~30 nm to several micrometers in diameter [1]. They are ubiquitously present in various body fluids, including blood, urine, cerebrospinal fluid, and saliva, and carry a rich cargo of proteins, lipids, mRNAs, and microRNAs. As key mediators of intercellular communication, EVs carry bioactive molecules such as proteins, lipids, mRNA, microRNA (miRNA), and DNA. They can be taken up by recipient cells through ligand–receptor binding, membrane fusion, or endocytosis, thereby modulating the function and behavior of the recipient cells [2].

EV biogenesis is a highly regulated endosomal pathway process. First, the plasma membrane invaginates to form early endosomes. Subsequently, early endosomes mature into late endosomes, during which inward budding of the limiting membrane generates intraluminal vesicles (ILVs). Vesicles containing abundant ILVs are referred to as multivesicular bodies (MVBs). Ultimately, MVBs fuse with the plasma membrane, releasing their internal ILVs into the extracellular space; these released ILVs are termed EVs [3].

Under physiological conditions, EVs are extensively involved in key processes such as immune regulation, tissue repair, angiogenesis, and neural signal transmission [4]. However, under pathological conditions, EV functions are frequently dysregulated and contribute to the onset and progression of various diseases. In oncology, EVs can promote tumor proliferation, angiogenesis, invasion, metastasis, and immune evasion [5]. In neurological disorders, EVs participate in axon regeneration and myelin repair following peripheral nerve injury [6], and are also associated with the pathological spreading of neurodegenerative diseases such as Alzheimer’s disease and Parkinson’s disease [7]. Furthermore, EVs play important roles in autoimmune diseases such as multiple sclerosis [8], cardiovascular diseases including myocardial infarction and heart failure [9], traumatic brain injury [10], diabetes, and inflammatory diseases [11].

Given their favorable biocompatibility, low immunogenicity, and potential as natural drug carriers, EV-based cell-free therapies are regarded as important directions in regenerative medicine and precision medicine [12]. However, the clinical translation of EV therapy faces a core bottleneck: extremely low yield. Traditional two-dimensional (2D) cell culture methods for EV production are inefficient and costly, failing to meet the demands for large-scale and standardized preparation [13,14].

Notably, the rapid advances in biosensing technologies are opening a new dimension for overcoming this bottleneck. On one hand, advanced detection platforms are no longer merely end-point analytical tools for EVs, but are progressively permeating the entire process management of EV production. For example, real-time sensors based on electrochemical impedance or surface plasmon resonance have been employed to continuously monitor EV release rates in cell culture supernatants, thereby providing immediate feedback for dynamically optimizing physical or biochemical stimulation parameters (e.g., mechanical loading frequency, electric field intensity, or drug treatment duration) and guiding the selection of optimal harvest windows. On the other hand, integrated microfluidic chips are demonstrating unique advantages in combining cell culture, stimulation intervention, on-line separation, and multiplexed detection into a single platform. Such “production–detection–quality control” integrated systems enable rapid assessment of particle size distribution, marker expression, and batch-to-batch consistency for each EV preparation, greatly enhancing the standardization of production workflows. Furthermore, ultra-sensitive single-particle detection technologies—such as nano-flow cytometry and single-molecule arrays—allow fine characterization of the functional states of EV subpopulations, including surface ligand activity, target cell uptake efficiency, and quantitative profiling of specific miRNA/protein cargos, all of which are critical for ensuring the potency and safety of therapeutic EVs. Therefore, the organic integration of biosensing technologies with the various secretion-enhancing strategies discussed in this review will not only deepen our understanding of the kinetic regulation of EV biogenesis, but also provide key technological support for ultimately achieving scalable and intelligent manufacturing of clinical-grade EVs.

**Figure 1:**
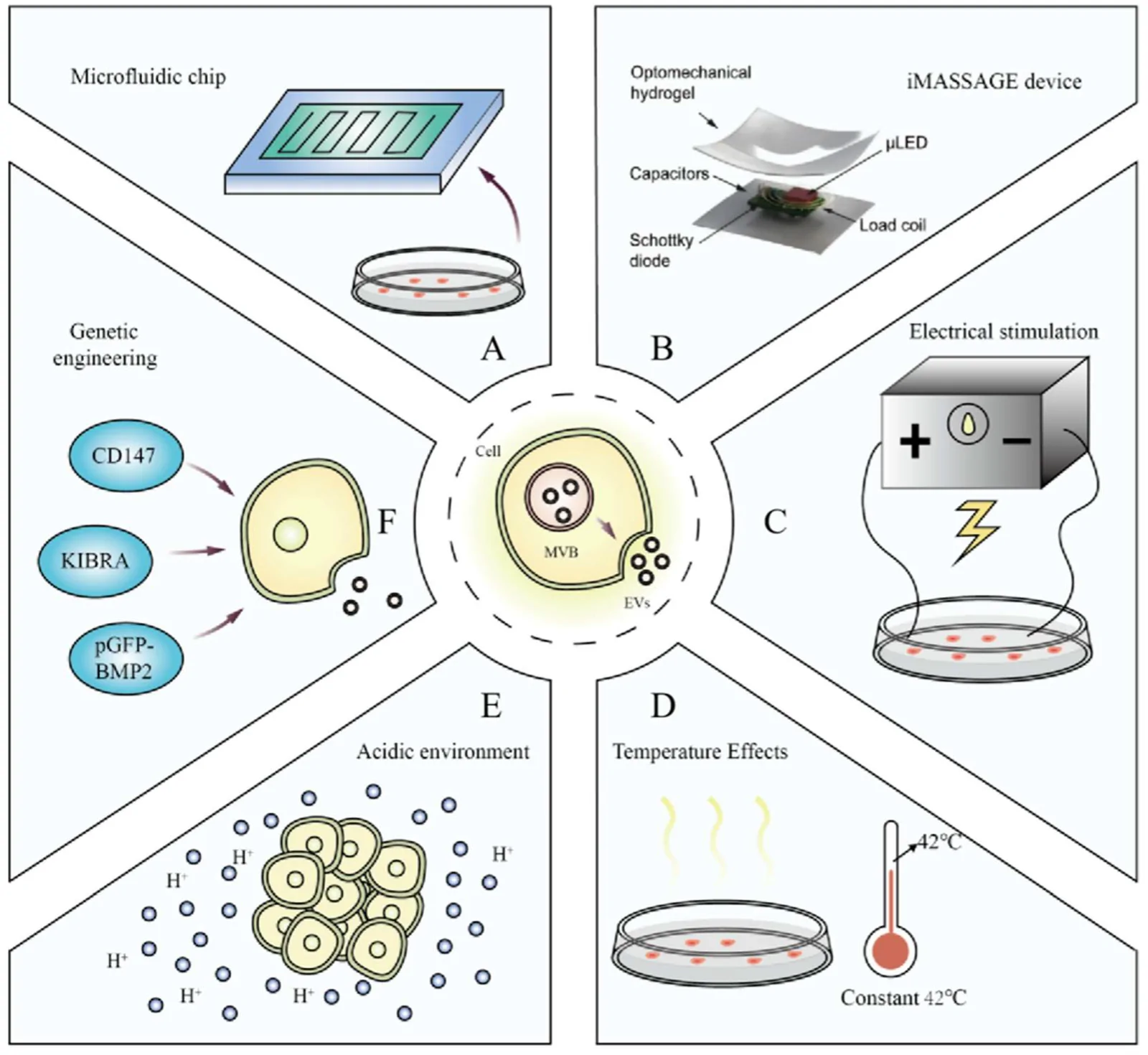
Physical and Biochemical Methods for Promoting EV Release. (**A**) Microfluidic chip promote EV release. (**B**) Mechanical force promote EV release. (**C**) Electrical stimulation promote EV release. (**D**) Thermal stimulation promote EV release. (**E**) acidic environment promote EV release. (**F**) Genetic engineering promote EV release.

To address this challenge, researchers have developed various strategies to promote cellular EV release. These methods can be broadly divided into two categories: physical and Biochemical approaches (Figure 1). Physical approaches include mechanical loading, geometric structure modulation, acoustic stimulation, and electrical stimulation; non-physical approaches include molecular interference, environmental factor regulation, and external inducers. These innovative strategies have been demonstrated to significantly increase EV yield, providing key technical support for advancing the clinical translation of therapy.

## 2. Physical Approaches for Enhanced EV Secretion

In this review, physical stimulation modalities are classified according to their primary energy input (mechanical, electrical, acoustic, optical, and thermal) [15]. Under this framework, ‘mechanical stimulation’ is defined broadly to include both directly applied physical forces (e.g., pressure, stretch, shear) and forces generated indirectly via energy conversion—such as magneto-mechanical transduction, where magnetic fields act on responsive nanomaterials to produce cellular deformation and traction. This unified definition ensures that all strategies that ultimately remodel the cytoskeleton through force transmission are consistently grouped under mechanical loading, regardless of the initial energy source. Thus, magnetic preconditioning is discussed here as a remote, non-contact implementation of mechanical stimulation.

### 2.1. Mechanical Loading Stimulation

Mechanical force, as an exogenous physical stimulus, has been proven to effectively promote EV biogenesis and secretion at multiple levels [16], serving as a key regulatory node connecting the mechanical microenvironment and vesicular transport. Whether it is direct mechanical pressure, osmotic pressure-induced changes in cell volume, magneto-mechanical transduction mediated by magnetic fields, or fluid shear stress in three-dimensional culture and microfluidic chips, the core mechanisms by which these seemingly different forms of physical stimulation promote EV release converge on a common pathway—namely, mechanical signal transduction-induced cytoskeletal remodeling, which in turn regulates the transport of multivesicular bodies (MVBs) and their fusion efficiency with the plasma membrane.

**Figure 2:**
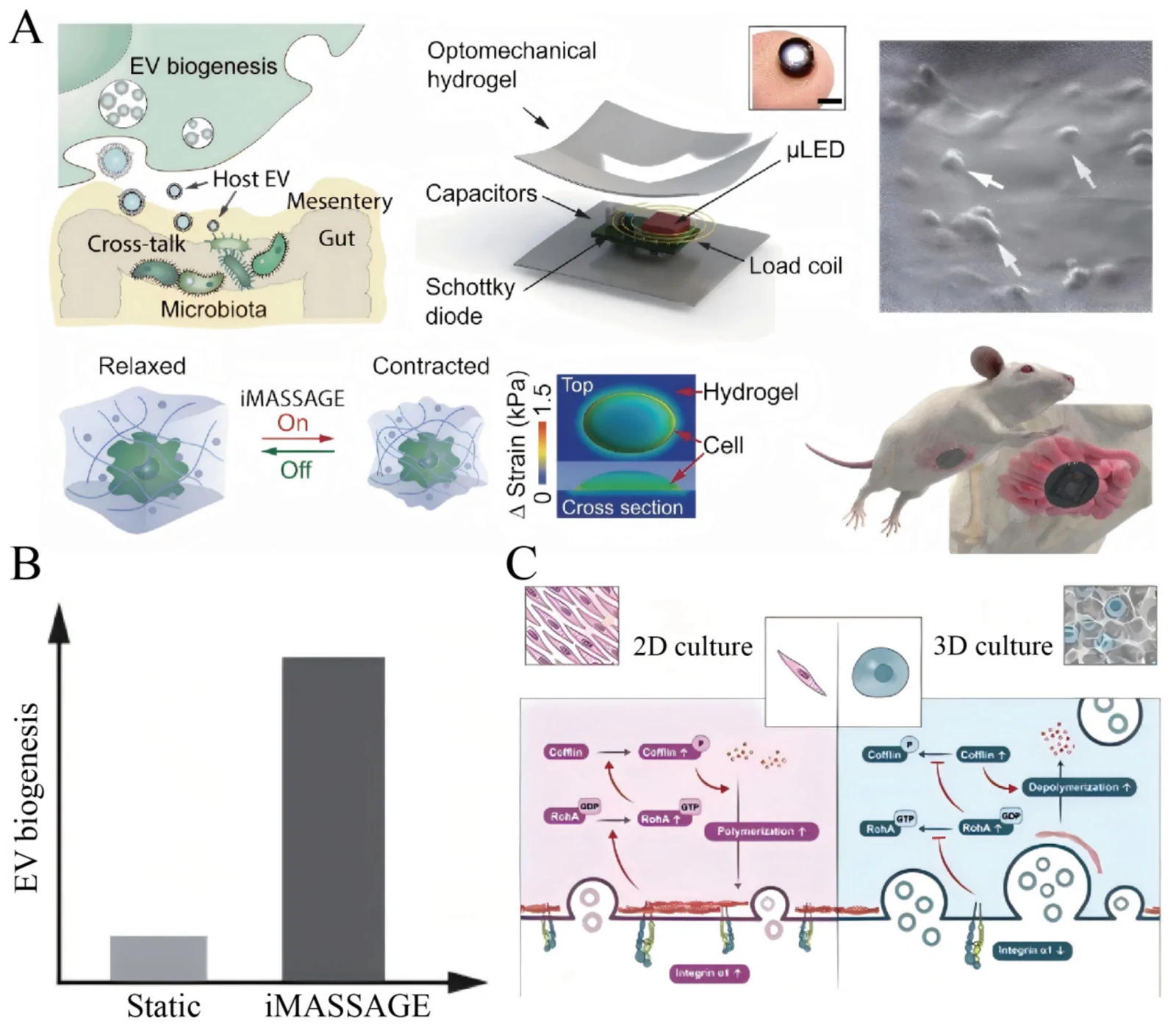
Increase EV production by using mechanical methods. (**A**) IMASSAGE for in vivo EV production and in situ microbiota modulation. Reproduced from Ref. [17], Copyright 2024, Springer Nature. (**B**) Compared to static cell culture conditions, iMASSAGE treatmentpromoted the production of EVs. Reproduced from Ref. [17], Copyright 2024, Springer Nature. (**C**) Schematic illustration depicting the potential mechanism underlying EV secretion byMSCs cultured under 3D conditions. Reproduced from Ref. [18], Copyright 2025, John Wiley and Sons.

At the in vivo level, Wan et al. constructed a miniature wireless bioelectronic system named iMASSAGE (Figure 2A), which can wirelessly trigger periodic volume contraction and relaxation of a hydrogel, applying pulsed mechanical stretch to embedded macrophages. This device increased the yield of therapeutic EVs by up to 20-fold (Figure 2B). When implanted into the mesentery of mice with inflammatory bowel disease, it achieved in situ, on-demand EV production and microbiota modulation [17]. This work demonstrated for the first time that controllable mechanical force stimulation can efficiently drive EV biogenesis within the complex in vivo physiological environment.

At the cellular level, Ji et al. utilized high spatiotemporal resolution two-color CD63 reporter gene live-cell imaging technology to observe in real-time at the single-vesicle level that mechanical pressure promotes the fusion of MVBs with the plasma membrane [19]. They found that mechanical pressure applied by a PDMS membrane increased EV production by approximately 2-fold. The mechanism involved pressure-induced reorganization of the actin cytoskeleton from a disordered distribution to regularly arranged stress fibers, which significantly enhanced the anchoring and fusion efficiency of MVBs at the plasma membrane. Additionally, mechanical pressure caused the clustering of SNAP-23 protein on the plasma membrane, further promoting MVB-plasma membrane fusion. Meanwhile, Li et al. found that hyperosmotic pressure induced cells to release extracellular vesicles containing mitochondrial components (MEVs), and regulated cellular reprogramming through activation of the TNF-α/β-catenin signaling axis [20]. These results indicate that both solid mechanical pressure and osmotic stress can enhance EV release by affecting the cytoskeleton and membrane fusion-related proteins.

Extending mechanical stimulation from transient intervention to a continuous culture strategy, three-dimensional (3D) culture systems systematically increase EV yield by mimicking the in vivo mechanical microenvironment. Compared with traditional two-dimensional culture, 3D culture can increase EV yield by approximately 2.1- to 16-fold. Huo et al. revealed that 3D culture reduces integrin β1 expression, leading to cortical actin depolymerization, while upregulating RAC1 to promote actin aggregation on MVB membranes, thereby synergistically enhancing EV biogenesis and secretion [21]. Yang et al. further found that 3D culture downregulates integrin α1, inhibits the RhoA/cofilin signaling pathway, and induces cortical actin depolymerization, and this process does not depend on the Rab27A/B regulatory pathway (Figure 2C) [18]. Wu et al. employed a 3D culture system based on gelatin methacryloyl (GelMA) hydrogel, demonstrating that this system not only delays the senescence of bone marrow mesenchymal stem cells (BMSCs) to maintain their secretory capacity, but also that the derived EVs are superior to those from 2D culture in promoting angiogenesis and full-thickness skin defect repair [22]. Collectively, these studies indicate that the continuous mechanical stimulation experienced by cells in 3D culture reshapes EV biogenesis and release efficiency through the integrin-cytoskeleton axis.

Magnetic field, as a non-contact physical stimulus, ultimately also exerts its promoting effect on EV secretion through mechanical force. Wu et al. used Fe_3_O_4_ nanoparticles combined with a static magnetic field (SMF) to precondition BMSCs. Under synergistic conditions of 50 μg/mL nanoparticles and 100 mT SMF, EV yield was significantly increased. The obtained EVs promoted the proliferation, migration, and tube formation of human umbilical vein endothelial cells (HUVECs), as well as the migration of human dermal fibroblasts in vitro. In a diabetic rat full-thickness skin defect model, local transplantation of these EVs accelerated wound healing and enhanced angiogenesis. Mechanistically, miRNA sequencing revealed that miR-21-5p was highly enriched in mag-BMSC-Exos, which activated the PI3K/AKT and ERK1/2 signaling pathways by targeting and inhibiting SPRY2 [23]. Subsequently, Wu et al. extended this strategy to the field of bone regeneration, finding that BMSC-Fe_3_O_4_-SMF-Exos significantly promoted the osteogenic differentiation of BMSCs and the migration and tube formation of HUVECs, leading to significant increases in new bone volume and vascular density in a rat calvarial defect model. Among these, miR-1260a played a dual regulatory role by targeting and inhibiting HDAC7 and COL4A2 [24]. It is noteworthy that magnetic fields do not directly participate in cellular biochemical reactions; their biological effects are primarily achieved through magneto-mechanical transduction. Under a static magnetic field, Fe_3_O_4_ nanoparticles experience magnetic forces, applying sustained micromechanical stretch to the cells. This physical pulling signal is transmitted into the cell through mechanoreceptors such as integrins, triggering cytoskeletal remodeling and signaling pathway activation similar to those induced by direct mechanical pressure. Therefore, magnetic field preconditioning is essentially a remote, non-contact mechanical stimulation strategy achieved through magneto-mechanical coupling, and its downstream YAP/TAZ mechanotransduction pathways and cytoskeletal dynamics are highly similar to those of direct mechanical loading.

At the engineering level, microfluidic chip technology achieves high-throughput EV production by optimizing the fluidic mechanical microenvironment. Huang et al. designed a PDMS microfluidic system integrating herringbone grooves and micropillar arrays. Mechanistically, the micropillar arrays operate on deterministic lateral displacement (DLD), where pillar geometry (gap G and tilt angle θ) defines a tunable critical diameter (Dc ≈ 1.4G·tanθ^0.48) for size-based EV isolation. Concurrently, the laminar flow splitting within the array generates shear stress that promotes EV biogenesis via mechanotransduction. Thus, the micropillar array serves dual functions: size-selective EV separation and mechanical stimulation for enhanced production. This design three-dimensional structure can induce turbulent vortices at a flow rate of 1 μL/min, simultaneously achieving uniform nutrient distribution and precise application of fluid shear stress. The study found that an intermittent perfusion strategy with a 12-h cycle more effectively stimulated the EV secretion potential of cells. Furthermore, this work combined genetic engineering with the microfluidic platform, overexpressing hepatocyte growth factor (HGF) in cells; the resulting EVs exhibited excellent pro-angiogenic and immunomodulatory effects in diabetic rat wound repair [25]. Fluid shear stress in microfluidics is also a form of mechanical stimulation: the wall shear stress generated by flow acts on the cell surface, is transduced through the integrin-cytoskeleton axis, and ultimately converges on the common pathway of MVB transport and plasma membrane fusion.

In summary, from implantable in vivo mechanical stimulation devices, to magneto-mechanical transduction mediated by magnetic fields, and to fluid shear stress regulation in 3D culture systems and microfluidic chips, the core mechanisms by which these seemingly diverse physical stimulation strategies promote EV release are highly unified—all physical signals ultimately converge on cytoskeletal remodeling as a key node through mechanotransduction pathways, thereby regulating MVB anchoring, transport, and plasma membrane fusion. Mechanical force stimulation directly remodels the cytoskeleton through the integrin-F-actin-YAP/TAZ axis; magnetic fields generate cellular traction forces through magneto-mechanical transduction of Fe_3_O_4_ nanoparticles, which are also transmitted by integrins and trigger similar cytoskeletal reorganization and signaling cascades. This convergent mechanical regulatory mode not only provides a unified theoretical framework for understanding how physical stimuli regulate vesicular transport, but also opens up diverse technological pathways for the large-scale production and functional enhancement of EVs.

### 2.2. Electrical Stimulation

Electrical stimulation, as an exogenous physical intervention, has been shown to effectively promote the biogenesis and secretion of extracellular vesicles (EVs) at multiple levels [26], acting as a key regulatory hub that links the electrical microenvironment with vesicle trafficking. From direct electric field stimulation and current transmission via conductive biomaterials to the piezoelectric and photopyroelectric effects at ferroelectric active interfaces, these various forms of electrical stimulation all activate intracellular calcium signaling pathways, modulate the activity of key enzymes, and remodel the cytoskeleton, ultimately converging on enhanced biogenesis of multivesicular bodies (MVBs) and their fusion with the plasma membrane.

A natural starting point for investigating how electrical stimulation influences EV secretion is the relationship between electric field parameters and cellular responses. Wang et al. developed a customized three-dimensional bioreactor capable of applying programmable electric field stimulation in an in vivo-like culture environment [27]. When a physiologically relevant electric field (5 mV/mm), comparable to that used in therapeutic deep brain stimulation, was applied, EV release and protein cargo changed in a frequency-dependent manner: low-frequency (2 Hz) fields increased intracellular vesicle mobility, whereas higher frequencies (200 Hz) preferentially upregulated proteins involved in mRNA processing. This finding points to an intrinsic link between the frequency of electrical stimulation and the efficiency of intracellular vesicle trafficking.

**Figure 3:**
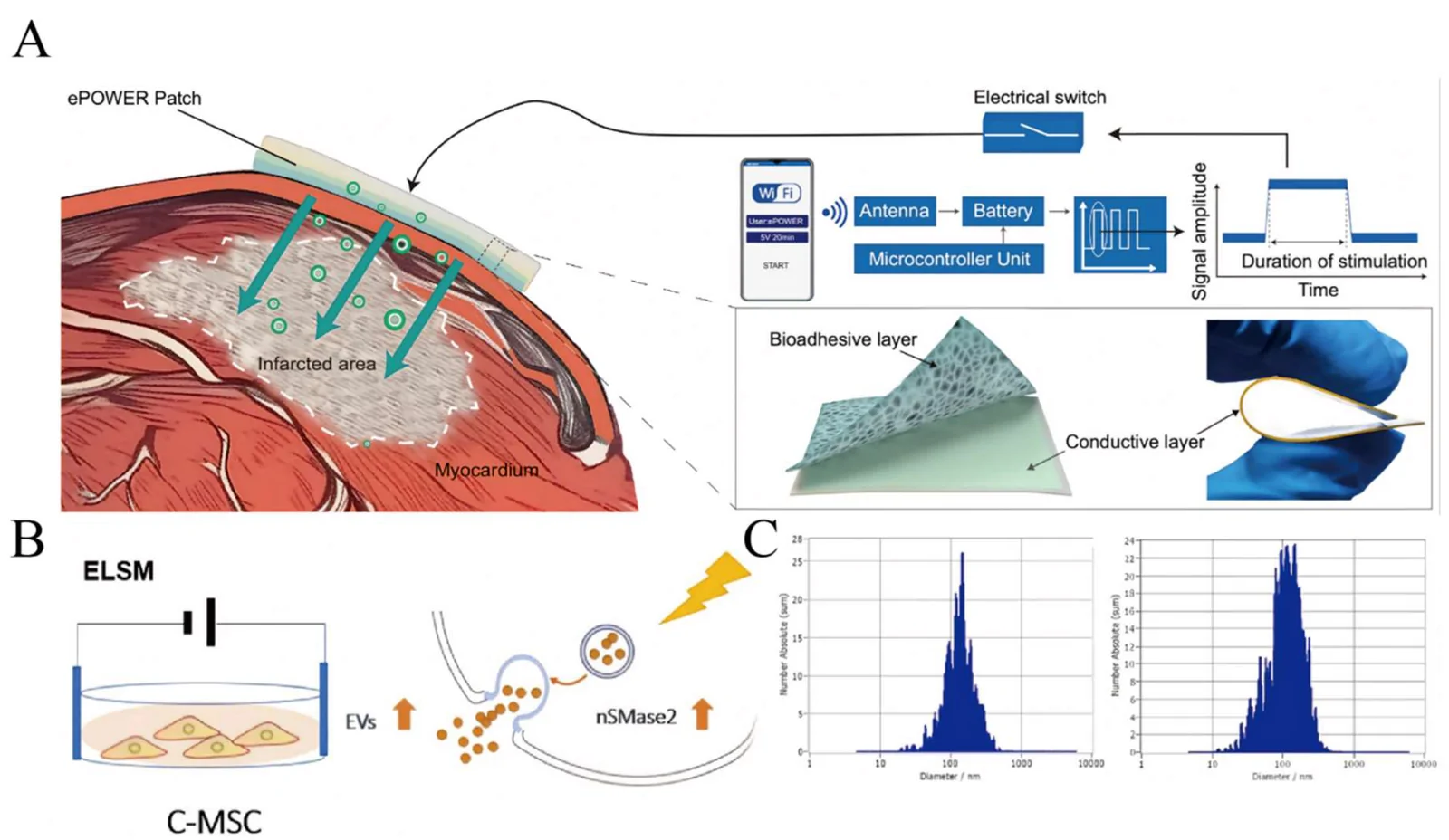
Schematic of electrical stimulation promoting EV release. (**A**) Design of the implanted ePOWER patch system for electromodulated therapeutic extracellular vesicle (EV) production and in-situ therapy. Being attached to the impaired heart, ePOWER patch induces BV2 macrophages under load to produce bioactive EVs that improve cardiac repair. ePOWER system integrates an adhesive electroactive patch, a microprogramming control unit (MCU) module, and a WiFi antenna module to enable smartphone control of the treatment process. Reproduced from Ref. [29], Copyright 2025, Springer Nature. (**B**) Schematic overview of proposed mechanisms of ELSM-induced cardiomyocyte protection. ELSM increases expression of nSMase2, leading to increased EV secretion. Reproduced from Ref. [28], Copyright 2023, MDPI. (**C**) nanoparticle tracking analysis using ZetaView^®^ Particle Tracking Analyzer to measure the size and number of C-MSC-derived EVs with or without ELSM. Reproduced from Ref. [28], Copyright 2023, MDPI.

Shifting the focus from physical parameters to molecular events, Zhang et al. directly connected electrical stimulation signals to the core enzymatic step of EV biogenesis [28]. They found that electrical stimulation (0.5 Hz, 1.5 V/1.8 cm) of cardiac mesenchymal stem cells markedly increased EV secretion (Figure 3B,C), and the resulting EVs conferred stronger hypoxia-induced anti-apoptotic protection to cardiomyocytes. Mechanistically, electrical stimulation upregulated the protein expression of neutral sphingomyelinase 2 (nSMase2) in a time-dependent manner—an enzyme that is crucial for intraluminal vesicle formation within MVBs. siRNA-mediated knockdown of nSMase2 significantly suppressed the stimulation-induced increase in EV secretion and abrogated the cardioprotective effect. A molecular pathway from electrical stimulation through nSMase2 to EV biogenesis was thus established.

Parallel to this enzymatic regulatory pathway, intracellular calcium signaling represents another key route through which electrical stimulation promotes EV secretion, and it works in close synergy with nSMase2. Fu et al. constructed a programmable wireless bioelectric active patch system called ePOWER (Figure 3A). Applying a 5 V direct current electric field to embedded M2-type BV2 microglia for 20 min increased EV production per cell by approximately 24-fold compared with unstimulated controls [29]. Mechanistic studies revealed that electrical stimulation induced a pronounced intracellular Ca^2+^ influx; the Ca^2+^ chelator EGTA drastically reduced EV yield, whereas direct addition of CaCl_2_ was sufficient to mimic the EV secretion-promoting effect. Transmission electron microscopy further confirmed that the number of MVBs containing more intraluminal vesicles increased markedly after stimulation. Consistently, Peng et al. exploited the photopyroelectric effect of a ferroelectric active interface. Under near-infrared irradiation (0.66 W/cm^2^), a transient photopyroelectric field of approximately 23 V was generated in mesenchymal stem cells, which also triggered a robust intracellular Ca^2+^ influx and led to an approximately 8-fold increase in EV production per cell [30]. Transcriptomic analysis revealed activation of multiple signaling pathways related to Ca^2+^ transport, influx, and homeostasis. Notably, Ca^2+^ signaling does not act in isolation—it can directly promote the directed trafficking of MVBs toward the plasma membrane and further amplify the effect by activating downstream MAPK/ERK and PI3K-Akt kinase cascades, thereby functionally complementing the nSMase2-mediated intraluminal vesicle budding process within MVBs.

Building on these mechanistic insights, researchers have further combined electrical stimulation strategies with functional biomaterials to achieve precise EV delivery and maximize therapeutic effects. Liu et al. integrated polyphenol-engineered kelp-derived EVs with conductive microneedles, which not only deliver EVs transdermally into deep wound tissue but also effectively transmit exogenous electrical stimulation signals [31]. They found that applying 400 mV electrical stimulation increased the cellular uptake efficiency of EVs by approximately 1.53-fold, and the combined treatment significantly promoted healing in a diabetic wound model. Similarly, Chen et al. applied a 150 μA electric field pretreatment to bone marrow mesenchymal stem cells on a conductive ITO glass substrate (1 h per day for 5 consecutive days); the collected EVs showed significantly enhanced yield and protein content [32]. Proteomic and transcriptomic analyses revealed enrichment of oxidative phosphorylation and PI3K-Akt pathways, and Rho-GTPase activation may be involved in this process. When these electrically preconditioned EVs were loaded into a chondroitin sulfate hydrogel and injected into a bone defect site, marked new bone formation was observed postoperatively.

It is worth noting that, despite the wide variation in stimulation parameters (frequencies from 0.5 Hz to 200 Hz, currents from 150 μA to 5 V, and durations from minutes to days), cell types (macrophages, mesenchymal stem cells, fibroblasts, etc.), and stimulation modalities (direct electric field, conductive substrates, ferroelectric materials, conductive microneedles) across these studies, the resulting EVs consistently maintained the typical cup-shaped morphology, characteristic marker proteins (CD63, Alix, TSG101, etc.), and similar cellular uptake efficiencies. This indicates that electrical stimulation primarily affects EV quantity without altering their fundamental biological properties, providing a strong safety assurance for clinical applications.

Taken together, the core mechanisms by which electrical stimulation promotes EV secretion can be summarized into three interrelated pathways. First, electrical stimulation directly activates voltage-gated calcium channels, triggering Ca^2+^ influx that promotes intraluminal vesicle formation within MVBs and their directed trafficking to the plasma membrane. Second, it upregulates nSMase2 expression, accelerating the conversion of sphingomyelin to ceramide—a key biochemical step in intraluminal vesicle budding within MVBs. Third, it activates the MAPK/ERK and PI3K-Akt signaling cascades, facilitating vesicle transport and plasma membrane fusion. These three pathways operate in a coordinated manner: Ca^2+^ signaling can directly influence MVB trafficking efficiency and can also further amplify the nSMase2-mediated vesicle budding effect by activating downstream kinase cascades, collectively forming a highly efficient signaling network. As a clean, controllable, programmable, and biosafe physical intervention, electrical stimulation holds broad promise for various tissue regeneration therapies, including myocardial infarction repair, bone regeneration, diabetic wound healing, and neurovascular remodeling.

### 2.3. Acoustic Stimulation

Acoustic stimulation, as a non-invasive physical intervention, can markedly enhance EV release from various cell types [33]. This effect is parameter-dependent and reproducible, and has been validated in both in vitro and in vivo models. Acoustic stimulation is easy to perform and has a favorable safety profile; fine-tuned regulation can be achieved by adjusting the frequency, intensity, and duration. Current evidence suggests that the core mechanism primarily involves a transient increase in cell membrane permeability, leading to a rapid influx of extracellular calcium ions, which subsequently activates the endosomal sorting complexes required for transport (ESCRT) pathway. This drives the formation of multivesicular bodies (MVBs) and their fusion with the plasma membrane, ultimately increasing EV production. This calcium signaling–ESCRT axis exhibits a high degree of consistency across different frequencies and cell types. Although ultrasound inherently produces mechanical vibration and fluid shear stress, the dominant and well-characterized pathway for enhanced EV secretion involves acoustic-induced calcium influx and subsequent ESCRT activation. Therefore, based on its primary energy modality, ultrasound is classified as acoustic stimulation rather than mechanical stimulation, ensuring consistency with the energy-based categorization framework adopted throughout this review.

Low-intensity ultrasound is the most extensively investigated approach for promoting EV release. Zheng et al. systematically screened ultrasound parameters using adipose-derived stem cells and found that specific low-intensity conditions could increase the number of EV particles and total protein content by several orders of magnitude without significantly affecting cell viability [34]. The mechanism similarly follows the calcium influx–ESCRT pathway. This study also provided preliminary evidence of enhanced wound healing-related functions of the stimulated EVs, but its primary contribution lies in establishing a set of reproducible process parameters.

**Figure 4:**
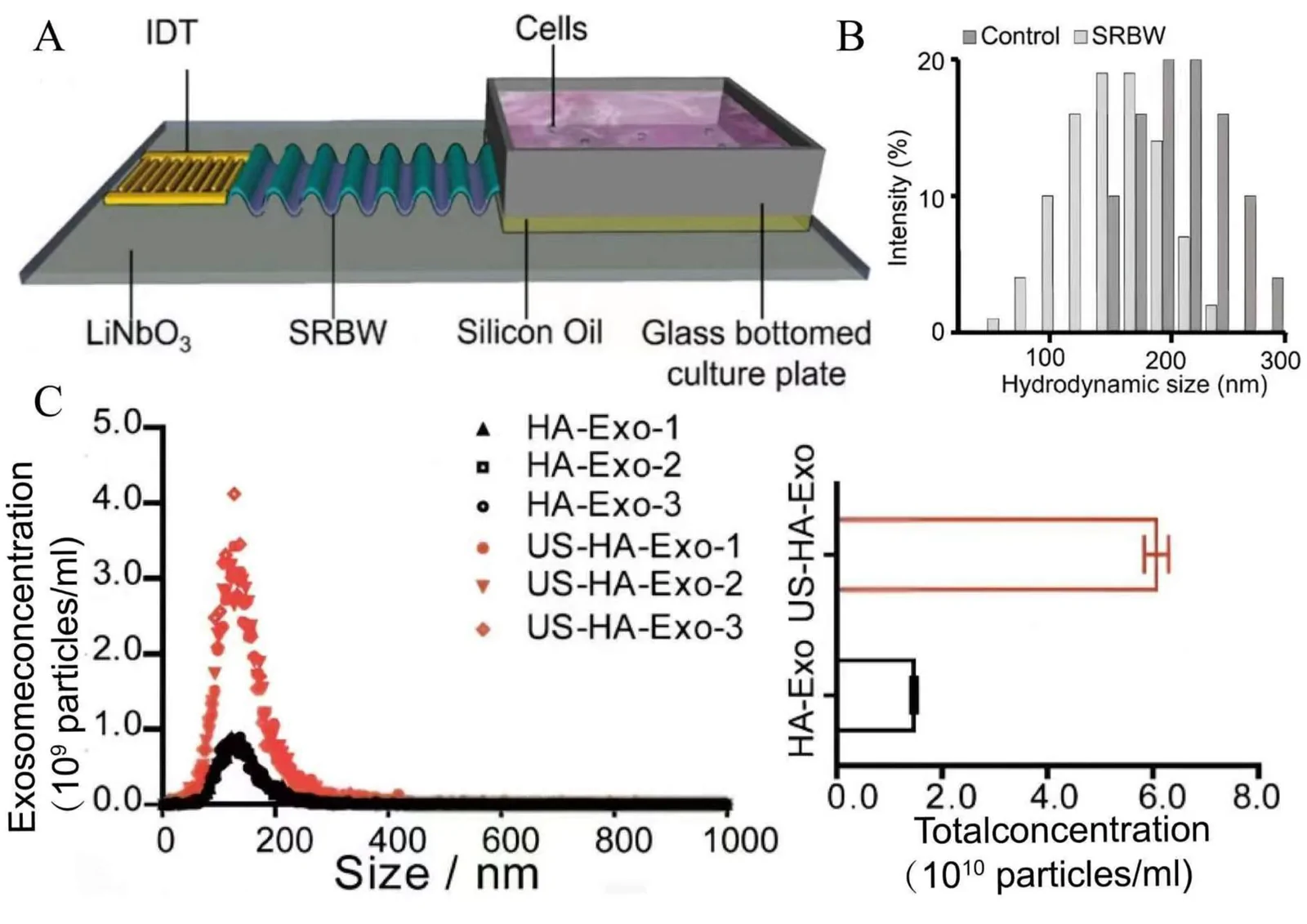
Use sound stimulation to boost EV release. (**A**) Perspective and side view schematics of the experimental set-up in which the SRBW (surface-reflected bulk waves) (not to scale), generated along a piezoelectric lithium niobate (LiNbO3) substrate by applying an AC electric signal at the device’s resonant frequency (10 MHz) to an interdigitated transducer electrode (IDT) photolithographically patterned on the substrate, is coupled through a thin layer of silicon oil into a glass-bottom culture plate containing the adherent cells to stimulate their production of EVs. Reproduced from Ref. [35], Copyright 2020, Springer Nature. (**B**) The hydrodynamic size distribution, obtained through DLS. Reproduced from Ref. [35], Copyright 2020, Springer Nature. (**C**) Nanoparticle tracking analysis (NTA) showed that ultrasound stimulation significantly increased the total number and total protein concentration (as verified by SDS-PAGE) of EVs released from astrocytes, without altering their size distribution. Reproduced from Ref. [36], Copyright 2020, Ivyspring International Publisher.

High-frequency acoustic stimulation can also efficiently drive EV production. Ambattu et al. applied megahertz-frequency acoustic waves to multiple cell lines and observed a marked increase in EV yield within half an hour after a single brief stimulation [35]. Because post-stimulation cell viability remained extremely high, the researchers devised a cyclic stimulation strategy, achieving several-fold to nearly ten-fold enrichment of total yield through multiple “stimulation–incubation” cycles. Mechanistic studies further clarified that high-frequency acoustic waves enhance membrane permeability, promote calcium influx, and activate the recruitment of ALIX and TSG101 in the ESCRT pathway, thereby driving MVB formation and release (Figure 4A–C). This provides a mechanistic rationale and a process protocol for the clinical application of high-frequency acoustic stimulation.

Combining acoustic stimulation with functional biomaterials can synergistically enhance its EV-promoting effect. Song et al. combined low-intensity pulsed ultrasound with a 3D-printed biodegradable zinc–copper alloy scaffold and found that the two synergistically promoted Schwann cell activation and EV secretion. After being taken up by bone marrow mesenchymal stem cells, these EVs enhanced their osteogenic differentiation capacity [37]. Although the study validated bone defect repair in an animal model, its highlight is the first integration of acoustic stimulation, metal ions, and the neuro-osteogenic coupling mechanism, offering a new concept for tissue regeneration.

At the in vivo level, focused ultrasound (FUS) combined with microbubbles can reversibly open the blood–brain barrier, providing technical support for brain-targeted EV delivery. Deng et al. found that ultrasound stimulation of astrocytes increased EV release by several fold (Figure 4C) [36], and these ultrasound-derived EVs could be efficiently internalized by neurons in vitro and attenuate amyloid-beta-induced cytotoxicity. In an Alzheimer’s disease mouse model, FUS treatment combined with these EVs significantly reduced brain plaques. Proteomic analysis indicated that proteins related to proteostasis were upregulated in the stimulated EVs. This study is the first to link acoustic stimulation-enhanced secretion with FUS-assisted delivery, constructing a complete technical chain from “production enhancement” to “delivery.”

Overall, from low-intensity ultrasound to high-frequency acoustic waves, and from single-cell stimulation to combinations with biomaterials and focused ultrasound delivery, the effect of acoustic stimulation in promoting EV release has been systematically validated in multiple models. The underlying mechanism is highly unified: acoustic wave-induced membrane perturbations promote calcium influx, which in turn activates the ESCRT pathway, regulating MVB formation and release. Acoustic stimulation offers controllable parameters and convenient operation, and can achieve large-scale EV production through cyclic stimulation or integration into culture systems, providing an adequate source guarantee for its clinical translation in regenerative medicine and drug delivery.

### 2.4. Light Stimulation

Light stimulation, as a novel physical induction strategy, has demonstrated unique advantages in promoting the efficient release of EVs in recent years. Compared with other stimulation modalities, light stimulation offers simple operation, low cost, remote spatiotemporal control, and ease of scalable integration, providing a highly promising solution to the core bottleneck of insufficient yield in the clinical translation of EVs. The underlying mechanism involves using specific wavelengths of light energy to apply controlled physical stimulation to donor cells, thereby activating intracellular signaling pathways—such as the oxidative stress pathway—to regulate key steps in EV biogenesis and secretion, achieving a substantial increase in yield without significantly compromising cell viability or product quality.

In terms of specific mechanistic investigation, Ruan et al. optimized the light wavelength, intensity, and irradiation time, and found that treating dendritic cells with 365 nm near-ultraviolet LED light (0.096 W/cm^2^, 30 min irradiation) increased the yield of EVs derived from immature and mature dendritic cells by approximately 13.3-fold and 5.1-fold, respectively [38]. The obtained EVs were highly consistent with their natural counterparts in particle size, morphology, and marker expression. Wu et al. constructed a photoswitchable mesenchymal stem cell system based on upconversion nanoparticles (UCNPs) [39]. By using 980 nm near-infrared light to excite intracellular UCNPs, localized 365 nm ultraviolet light was generated, which then activated the ROS/HEXB/LAMP1 signaling cascade, inhibited the fusion of lysosomes with multivesicular bodies (MVBs), and successfully increased EV yield by 2.7-fold. Both studies confirmed that the moderate oxidative stress induced by light stimulation did not impair cell viability, and the resulting EVs maintained good biological function and biocompatibility both in vitro and in vivo.

These results indicate that light stimulation technology is not only easy to perform and compatible with existing culture systems, but can also greatly enhance EV production while preserving their intrinsic quality. The underlying mechanism primarily relies on light-induced reprogramming of signaling pathways—such as the inhibition of lysosome–MVB fusion mediated by ROS—opening new avenues for the large-scale production and clinical translation of EVs.

### 2.5. Temperature Stimulation

Temperature, as a physical stimulus, has been demonstrated in multiple studies to significantly promote EV release from various cell types. The underlying mechanisms involve the activation of heat shock proteins, the upregulation of specific small GTPases (Rab family proteins), and the transcriptional reprogramming of genes related to EV biogenesis. This temperature-dependent regulatory strategy not only provides new bioengineering approaches for optimizing large-scale EV production, but also opens important perspectives for understanding the fundamental regulatory principles of EV biology.

At the mechanistic level, the promoting effect of temperature stimulation on EV release is primarily achieved through the regulation of Rab family small GTPases. Xu et al. found that in doxorubicin-resistant breast cancer cells MCF-7/ADR, hyperthermia treatment at 45 °C for 30 min significantly promoted EV secretion. Through RNA microarray analysis and subsequent validation, they identified Rab7b as the key molecule mediating hyperthermia-induced EV secretion—both the mRNA and protein levels of Rab7b were significantly upregulated after hyperthermia, and knocking down Rab7b markedly reduced EV secretion, confirming that hyperthermia drives EV release through the Rab7b molecular switch [40]. Correspondingly, Liao et al. raised the culture temperature of mast cells from 29 °C to 41 °C and observed a temperature-dependent increase in EV concentration. RNA sequencing revealed that Rab3a was a key gene in the vesicle secretion pathway; high temperature significantly upregulated its expression, and knocking down Rab3a markedly decreased EV secretion [41]. Although these two studies involve different cell types and Rab members, they collectively point to the central role of Rab GTPases in mediating temperature-promoted EV release.

At the multi-omics level, Keysberg et al. studied HEK293F suspension cells and found that mild hyperthermia treatment at 40 °C for 72 h increased the EV secretion per cell by approximately 6-fold. Integrated transcriptomic and proteomic analyses revealed that heat shock proteins (especially HSP70 and HSP90) and various components of the ESCRT complex were significantly upregulated under high-temperature conditions. Among them, HSP90 has been shown to directly mediate the fusion of multivesicular bodies with the plasma membrane, thereby promoting EV release [42]. Moreover, overexpression of ESCRT pathway genes such as CHMP1A, CHMP3, VPS28, and CD82 significantly increased EV titers, suggesting that temperature stimulation systematically enhances secretory capacity by coordinately upregulating the entire EV biogenesis pathway. Rayamajhi et al. further supplemented the evidence from the perspective of the interaction between culture duration and temperature, finding that K7M2 cells produced significantly higher exosomal protein yields under heat stress at 40 °C compared to 37 °C, and that the protein profiles of EVs differed markedly under different temperature conditions: proteins enriched under 40 °C heat stress were mainly involved in metabolic processes, whereas those enriched under 72 h nutrient stress conditions were associated with oxidative stress response and protein folding [43].

Taken together, temperature stimulation promotes EV release through a complex biological process involving the synergistic action of multiple pathways and targets, including the upregulation of vesicle trafficking regulators such as Rab7b and Rab3a, the activation of the heat shock protein network, and the transcriptional reprogramming of EV biogenesis-related genes such as those in the ESCRT pathway. This mechanism has been validated across different cell types, demonstrating a certain degree of universality. Notably, EVs produced under temperature stimulation also exhibit corresponding changes in their protein profiles and functional properties, indicating that attention must be paid to the consistency of product quality when utilizing temperature to optimize EV yield. These findings provide a clear theoretical basis and technical path for enhancing the production of therapeutic EVs through bioprocess optimization and cell engineering approaches, and hold significant reference value for the clinical translation of EV-based therapies.

In summary, although physical stimuli such as mechanical, electrical, acoustic, light, and thermal stimulation differ in their forms, they all converge on the core mechanism of regulating multivesicular body (MVB) trafficking and plasma membrane fusion, thereby significantly enhancing extracellular vesicle (EV) yield, with fold increases ranging from several-fold to over 20-fold. To facilitate a direct cross-comparison of the mechanisms and enhancement magnitudes among these strategies, we have summarized the representative studies in Table 1.

## 3. Biochemical Approaches for Enhanced EV Secretion

### 3.1. Genetic Engineering

Genetic engineering offers a precise and efficient strategy for modulating EV release. By directly intervening in key molecules involved in EV biogenesis and secretion pathways [44], it is possible to substantially increase EV yield and optimize their functional properties. Current approaches can be categorized into three levels of increasing technical complexity: (1) transgenic overexpression of positive regulatory factors; (2) engineering of ESCRT and Rab family components; and (3) gene editing using CRISPR/Cas9. This hierarchical framework enables a clearer understanding of the distinct mechanistic interventions and their translational potentials.

**Figure 5:**
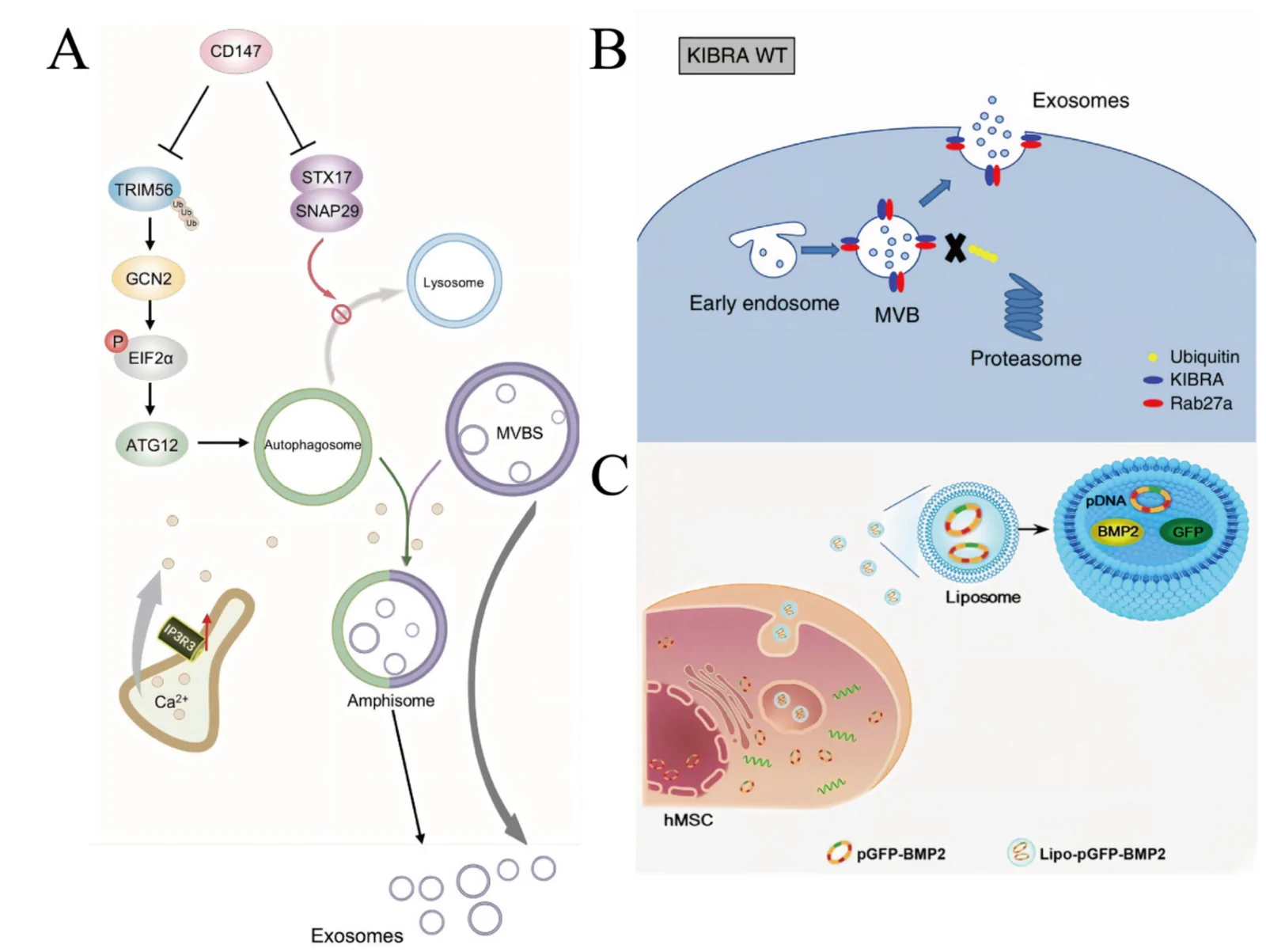
Promoting EV release through genetic engineering methods. (**A**) The proposed model of CD147 promotes EVs release through activation of crinophagy, thus facilitating tumor metastasis. CD147-overexpressing tumor cells establish a calcium-enriched intracellular niche by upregulating IP3R3 that potentiates amphisome biogenesis through enhanced autophagosome-MVBs fusion. This oncoprotein orchestrates a dual regulatory mechanism in autophagy flux: facilitating autophagosome assembly via TRIM56 suppression-mediated GCN2/EIF2α/ATG12 pathway activation, while simultaneously impeding autophagosome-lysosome docking through VAMP8/STX17/SNAP29 downregulation. Reproduced from Ref. [45], Copyright 2022, Springer Nature. (**B**) A schematic representation of a model for the role of KIBRA in the regulation of EV secretion. Reproduced from Ref. [46], Copyright 2019, Springer Nature. (**C**) A schematic illustration of genetically engineering hMSCs by liposome mediated BMP2 gene delivery. Reproduced from Ref. [47], Copyright 2025, Springer Nature.

Transgenic overexpression of positive regulators represents the most straightforward strategy. The tumor-associated antigen CD147 drives autophagosome assembly by activating the GCN2/EIF2α/ATG12 signaling axis, while simultaneously blocking VAMP8/STX17/SNAP29-dependent autophagosome-lysosome fusion, thereby promoting the fusion of autophagosomes with multivesicular bodies (MVBs) to form amphisomes and ultimately enhancing EV secretion (Figure 5A). In non-small cell lung cancer, CD147 overexpression is significantly positively correlated with EV release, and CD147-driven EV secretion can accelerate tumor metastasis [45]. Complementarily, the scaffold protein KIBRA stabilizes the small GTPase Rab27a by directly binding to it and inhibiting its ubiquitin-proteasomal degradation, thereby promoting the anchoring and fusion of MVBs with the plasma membrane. Functional experiments have shown that KIBRA knockdown significantly reduces EV secretion, whereas KIBRA overexpression enhances EV release (Figure 5B) [46]. Furthermore, modifying the gene expression profile of donor cells can also alter the cargo composition of EVs. After liposome-mediated transfection of the bone morphogenetic protein 2 (BMP2) gene into mesenchymal stem cells (MSCs), the mRNA levels of osteogenic differentiation-related genes (Runx2, OSX, ALP, etc.) were significantly upregulated in the donor cells, and these osteogenesis-related mRNAs were also enriched in the secreted EVs, thereby exhibiting significantly enhanced bone regeneration effects both in vitro and in vivo (Figure 5C) [47]. This study also found through plasmid tracking that a portion of the EVs could carry the plasmid DNA introduced into the donor cells and deliver it to recipient cells, suggesting that genetically engineered EVs have the potential to directly deliver therapeutic nucleic acids. Of note, the observed osteogenic activity may be attributed to both the BMP2 mRNA cargo and the co-packaged plasmid DNA, as both have been detected in the EVs; however, their relative contributions to the bone-regenerative effect have yet to be determined.

Engineering ESCRT complexes and their associated proteins represents a second, more sophisticated layer of regulation. Colombo et al. conducted a systematic RNAi screen targeting 23 ESCRT components and accessory proteins and found that different ESCRT members exhibit significant heterogeneity in regulating EV secretion [48]. Specifically, knockdown of the ESCRT-0 components HRS and STAM1 or the ESCRT-I component TSG101 reduced the secretion of CD63- and MHC II-positive EVs, whereas knockdown of VPS4B unexpectedly enhanced EV secretion without affecting EV morphology or size. More intriguingly, knockdown of the accessory protein ALIX increased intracellular MHC II mRNA and protein levels, but its effect on the total secretion of CD63-positive EVs was not significant and appeared cell-type dependent. Importantly, further analysis indicated that ALIX primarily influences EV cargo composition rather than the overall release efficiency. In HeLa cells, ALIX knockdown promoted the enrichment of MHC II into EVs, whereas this cargo-loading effect was not observed in human dendritic cells. These findings reveal the complexity of ESCRT component functions across different cellular contexts and provide a theoretical basis for finely tuning EV yield and composition by targeting specific ESCRT members. Beyond knockdown approaches, overexpression of specific ESCRT-related genes has also been validated. Keysberg et al. demonstrated that simultaneous overexpression of six genes—CHMP1A, CHMP3, CHMP5, VPS28, CD82, and EZR—in HEK293F suspension cells significantly increased the secretion titer of small extracellular vesicles, further confirming the ESCRT pathway as a tractable engineering target for yield enhancement [38].

Gene editing via CRISPR/Cas9 constitutes the most advanced and precise toolset. The introduction of this technology has further expanded the application boundaries of engineered EVs. It can be used to knock out genes involved in EV biogenesis to dissect their functions, and also enables stable overexpression of therapeutic cargo in donor cells, ensuring that the secreted EVs consistently carry specific effector molecules [47]. In stem cell engineering, the strategy of transiently transfecting MSCs with the BMP2 gene (as described above) avoids the risk of insertional mutagenesis associated with viral vectors, while allowing the donor cells to serve as “cell factories” that continuously produce EVs with enhanced therapeutic efficacy. This approach ingeniously confines the potential toxicity of gene delivery to the donor cell level; even when liposomal transfection reagents cause significant cytotoxicity to the donor cells, the ultimately secreted EVs exhibit excellent biocompatibility and have no toxic effect on recipient cells [47]. While direct applications of CRISPR/Cas9 specifically to boost EV yield are still emerging, its integration with donor cell engineering holds great promise for creating customized EV-producing cell lines with precisely controlled secretion profiles.

In summary, genetic engineering achieves precise intervention at key nodes of the EV biogenesis and secretion pathways, substantially increasing EV yield. The underlying regulatory effects can be clearly classified into two main axes. First, promoting MVB–plasma membrane fusion: the scaffold protein KIBRA stabilizes the small GTPase Rab27a by directly binding to it and inhibiting its ubiquitin-proteasomal degradation, thereby enhancing MVB docking and fusion at the plasma membrane. Second, suppressing MVB diversion toward lysosomal degradation: the tumor-associated antigen CD147 drives autophagosome assembly via the GCN2/EIF2α/ATG12 axis while simultaneously blocking VAMP8/STX17/SNAP29-dependent autophagosome-lysosome fusion, thus promoting autophagosome–MVB fusion to form amphisomes and redirecting MVBs toward secretory release. In addition, differential modulation of ESCRT components (e.g., HRS, TSG101, VPS4B, ALIX) reveals cell-type-dependent complexity: some members primarily affect ILV formation (e.g., HRS, TSG101), whereas others (e.g., VPS4B) may influence MVB trafficking or the degradation-secretion balance. Collectively, these strategies demonstrate that enhancing EV output can be achieved either by boosting MVB biogenesis and secretory capacity at the synthetic end, or by blocking lysosomal clearance at the degradative end. Moreover, co-regulation can endow EVs with specific therapeutic cargos (e.g., BMP2 mRNA), laying a technical foundation for engineering “high-yield and high-function” EVs.

### 3.2. Small Molecule Compound and Chemical Drug Intervention

Small molecule compounds and chemical drugs, as exogenous pharmacological tools, have demonstrated strong interventional potential in the study of EV release regulation [49]. Unlike physical stimuli that act indirectly through endogenous pathways, chemical drugs can directly target key nodes in ion channels, lysosomal acidification, and the unfolded protein response (UPR), enabling precise and quantifiable modulation of EV release. To date, a multi-target and reversible chemical toolbox for regulating EV release has been established, ranging from calcium signaling activation mediated by the ionophore monensin, to the endoplasmic reticulum (ER) stress–lysosomal acidification regulatory axis triggered by thapsigargin, and further to supplementary interventions by lysosomal disruptors such as chloroquine and bafilomycin A1. This has provided an important foundation for mechanistic dissection and translational applications.

Calcium signaling is a core node in the regulation of EV release, and the ionophore monensin is the most thoroughly studied secretagogue compound. Savina et al. found in K562 cells that monensin elevates intracellular Na^+^ through Na^+^/H^+^ exchange, which in turn reverses the Na^+^/Ca^2+^ exchanger, leading to a sustained increase in cytosolic Ca^2+^ and significantly stimulating EV release; this effect could be completely blocked by the intracellular Ca^2+^ chelator BAPTA-AM [50]. Moreover, monensin induced the accumulation of large amounts of Ca^2+^ inside multivesicular bodies (MVBs), forming giant Ca^2+^-enriched MVBs, suggesting that intra-luminal Ca^2+^ may directly participate in the fusion process. Guo et al. demonstrated that monensin enhanced EV release in a dose-dependent manner in prion-infected neurons and renal epithelial cells, and that the elevated EV secretion directly promoted the intercellular spread of pathological proteins. Conversely, blocking EV release with the neutral sphingomyelinase inhibitor GW4869 attenuated prion transmission, further supporting the role of EVs in propagation [51].

The functional coupling between ER stress and lysosomal acidification represents another important regulatory pathway. Zhou et al. discovered in melanoma cells that thapsigargin inhibits the SERCA pump, leading to ER Ca^2+^ depletion and activating the PERK and IRE1α branches of the UPR [52]. PERK, through its downstream transcription factor ATF4, inhibits the recruitment and assembly of V-ATPase to the lysosomal membrane, thereby reducing the degree of lysosomal acidification and catabolic activity; IRE1α similarly negatively regulates lysosomal function through an analogous mechanism. When PERK and IRE1α are inactivated, lysosomal acidification is enhanced, and the fusion of MVBs with lysosomes increases, resulting in MVB degradation rather than release; however, EV secretion can be restored by blocking lysosomes with the V-ATPase inhibitor bafilomycin A1. This finding reveals the logic that UPR signaling determines the “degradation or secretion” fate of MVBs by modulating lysosomal acidification.

Additionally, compounds that alter organelle pH also exhibit secretagogue activity. Savina et al. [50] found that chloroquine elevates cytosolic Ca^2+^ and stimulates EV release, an effect that could be blocked by the extracellular Ca^2+^ chelator EGTA; bafilomycin A1 similarly promotes EV secretion in a manner dependent on the presence of extracellular Ca^2+^. The combined use of chloroquine and monensin showed no additive effect, suggesting they may share overlapping mechanisms. The study by Zhou et al. further supplemented the explanation from the perspective of lysosomal acidification: bafilomycin A1 directly inhibits V-ATPase activity, preventing the diversion of MVBs toward lysosomal degradation.

Beyond traditional pharmacological agents, nanomaterial-based approaches have also emerged as effective modulators of EV secretion. Zhang et al. developed IGNITE, a ginseng-derived nanoparticle decorated with nanohydroxyapatite, which boosted dendritic cell EV secretion by 30-fold through intracellular calcium signaling, thereby amplifying tumor antigen presentation and synergizing with radiotherapy [53]. This example illustrates how nanoscale materials can serve as bioactive macromolecule surrogates or delivery vehicles to achieve potent and targeted EV yield enhancement, further expanding the chemical toolbox for EV production engineering.

In summary, small molecule compounds promote EV release through multi-target interventions—monensin activates Ca^2+^ signaling, thapsigargin triggers UPR–lysosomal acidification regulation, and chloroquine and bafilomycin A1 perturb organelle pH—all converging on the same outcome. These chemical tools not only provide powerful probes for uncovering the molecular mechanisms of EV biogenesis, but also lay an important foundation for pharmacologically enhancing EV production or intervening in pathological propagation. In the future, further screening of novel agonists with high specificity and low toxicity, and exploration of their synergistic effects with physical stimuli, are expected to advance the clinical translation of EV-based technologies.

### 3.3. Bioactive Macromolecules

**Figure 6:**
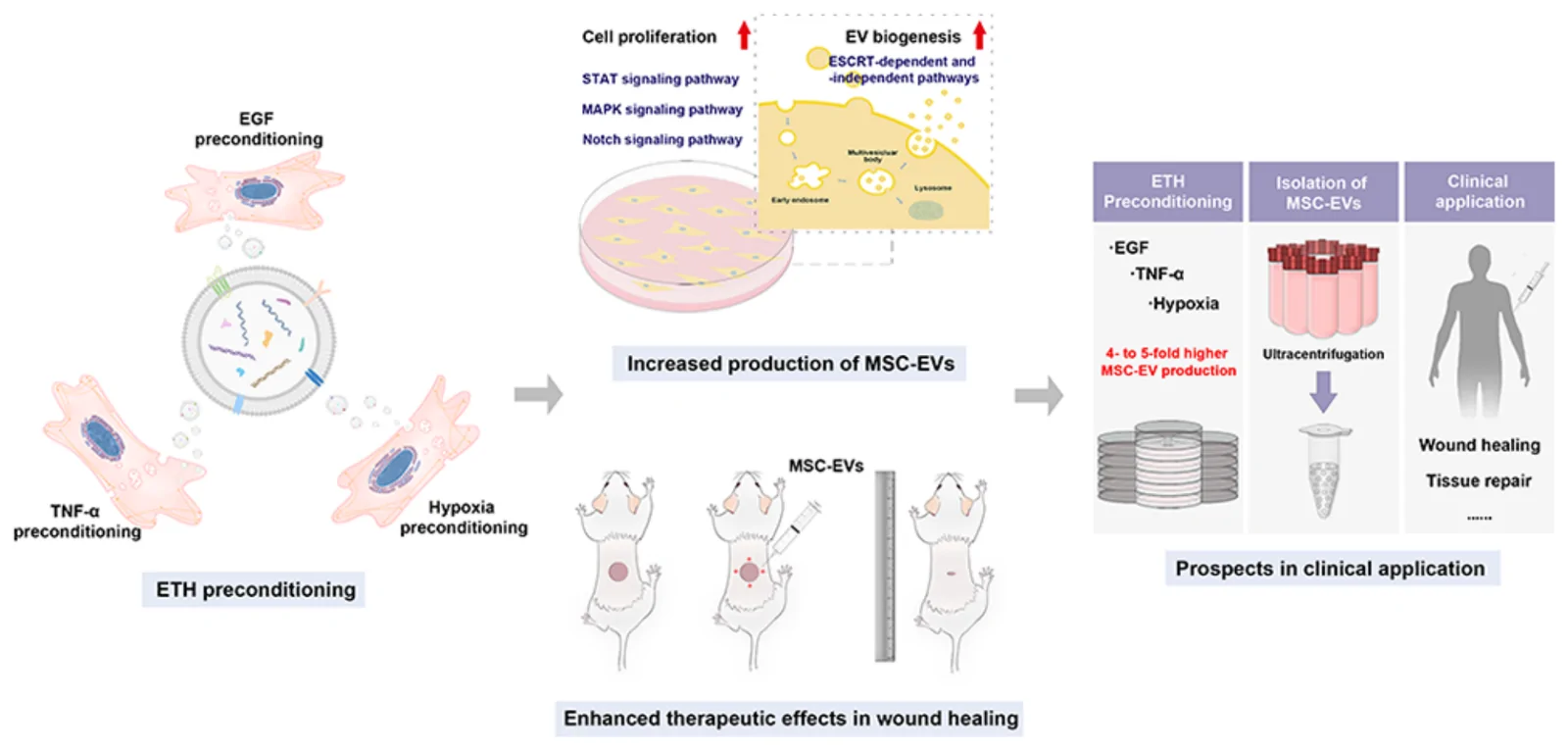
Bioactive macromolecules such as EGF and TNF-α enhance EV release. Red arrows indicate up-regulation. Reproduced from Ref. [54], Copyright 2025, American Chemical Society.

Bioactive macromolecules, as exogenous stimulation tools, have demonstrated strong interventional potential in regulating EV release from mesenchymal stem cells (MSCs). Unlike small molecule compounds that act through chemical interventions such as directly targeting intracellular ion channels, lysosomal acidification, and enzyme activities, bioactive macromolecules can specifically bind to cell surface receptors and activate downstream signaling cascades, thereby enabling precise reprogramming of donor cell functions. While enhancing EV yield, this strategy often also endows EVs with specific therapeutic functional cargos. To date, a multi-target and functionally designable regulatory strategy has been established, ranging from the synergistic activation of proliferation and secretion pathways by cytokines such as epidermal growth factor (EGF) and tumor necrosis factor-α (TNF-α), to the directed reinforcement of osteogenic differentiation pathways by bone morphogenetic protein 2 (BMP2) (Figure 6), to the remodeling of inflammatory microenvironment-responsive miRNA profiles by interleukin-1β (IL-1β), and further to the direct transcriptional regulation of core EV biogenesis genes by the transcription factor TEAD1. This has opened new avenues for the large-scale production and precision application of EVs.

Cytokine combination pretreatment is an effective strategy for achieving high EV yield and functional enhancement. Chen et al. systematically screened the effects of various biological, chemical, and physical stimuli on EV production from bone marrow mesenchymal stem cells (BMSCs) and found that EGF and TNF-α were the two most potent promoting factors, increasing the yield by 1.4-fold and 1.3-fold, respectively. Through orthogonal experimental optimization, they established a combined pretreatment regimen of EGF (10 ng/mL) and TNF-α (50 ng/mL), which significantly increased MSC-derived EV production. Proteomic analysis revealed that this combined treatment simultaneously activated cell proliferation-related signaling pathways such as Notch, MAPK, and STAT, as well as EV biogenesis pathways involving both ESCRT-dependent and -independent mechanisms, thereby achieving a dual enhancement of “proliferation promotion” and “secretion promotion” while maintaining MSC stemness [54].

Pretreatment with specific growth factors provides a precisely targeted strategy for the functionalization of EVs. He et al. found that EVs derived from BMSCs pretreated with bone morphogenetic protein 2 (BMP2), termed BMP2-Exos, exhibited significantly enhanced osteogenic effects in a diabetic microenvironment, while simultaneously suppressing inflammation through the induction of M2 macrophage polarization. Mechanistically, BMP2-Exos improved the impaired osteogenic function of diabetic BMSCs by upregulating the PI3K/AKT signaling pathway [55]. This study offers a safe and effective alternative that circumvents the adverse effects of direct in vivo BMP2 application, such as ectopic ossification and inflammation, through a “cell pretreatment–EV delivery” approach.

Inflammatory cytokine pretreatment can induce EVs to carry specific miRNA cargos, achieving synergistic anti-inflammatory and tissue regenerative functions. Chen et al. pretreated human umbilical cord mesenchymal stem cells (HucMSCs) with IL-1β, which not only significantly increased EV yield and protein content but also, through miRNA sequencing, revealed that IL-1β-induced EVs (C-Exos) were enriched with inflammation-regulatory miRNAs such as miR-205-5p and miR-149-5p, as well as matrix regeneration-related miRNAs like miR-766-3p. These miRNAs endowed C-Exos with synergistic anti-inflammatory and pro-chondrogenic regenerative functions by targeting key signaling pathways such as MAPK and PI3K-Akt [56]. This strategy suggests that inflammatory microenvironment signals can be “memorized” in the exosomal cargo, enabling functional customization tailored to specific pathological states.

Upstream regulation by transcription factors provides a novel molecular switch strategy for fundamentally enhancing EV yield. Pu et al. discovered that the transcription factor TEAD1 can serve as a molecular switch by directly binding to and activating the promoter regions of key genes (RAB11, CD9, and SNAP23) involved in EV trafficking, anchoring, and release, thereby significantly promoting EV biogenesis and secretion at the transcriptional level. Overexpression of TEAD1 in both adipose-derived and bone marrow mesenchymal stem cells markedly enhanced EV secretion without affecting the basic morphology or size of the EVs [57]. Notably, TEAD1 overexpression did not significantly alter the overall protein composition of the EVs, suggesting that TEAD1 primarily acts to boost EV production rather than change their basic protein makeup. However, this conclusion is limited to the proteomic level, as other cargo components—including miRNAs and lipids—were not examined in this study. Therefore, the observed consistency in protein composition supports the potential for achieving high yield with consistent protein profiles, but the uniformity of other EV cargoes remains to be further characterized.

In summary, bioactive macromolecules constitute a multi-target, multi-level regulatory system that is both functionally programmable and yield-enhancing. Their pro-secretory effects also converge on the core MVB fate-determining pathways. On one hand, they directly promote MVB–plasma membrane fusion: the transcription factor TEAD1 acts as a molecular switch by binding to and activating the promoters of key genes (RAB11, CD9, SNAP23), thereby transcriptionally enhancing MVB trafficking, anchoring, and fusion. Cytokine combinations (e.g., EGF and TNF-α) synergistically activate MAPK, STAT, and Notch signaling to boost donor cell proliferation and secretory capacity, indirectly increasing MVB synthesis and release flux. On the other hand, certain factors can functionally tailor EV cargo without compromising yield—for example, BMP2 pretreatment enriches osteogenic mRNAs via PI3K/AKT upregulation, while IL-1β pretreatment loads EVs with inflammation-modulatory miRNAs (e.g., miR-205-5p, miR-149-5p). Although these cargo-remodeling effects are not strictly degradative, their accompanying yield enhancement often relies on the same MVB generation and release pathways. Thus, bioactive macromolecules not only provide powerful probes for dissecting the regulatory networks of EV biogenesis, but also offer a viable route for producing therapeutically functional EVs through donor cell engineering.

### 3.4. Regulation by the Extracellular Microenvironment and Nutrient Stress

**Figure 7:**
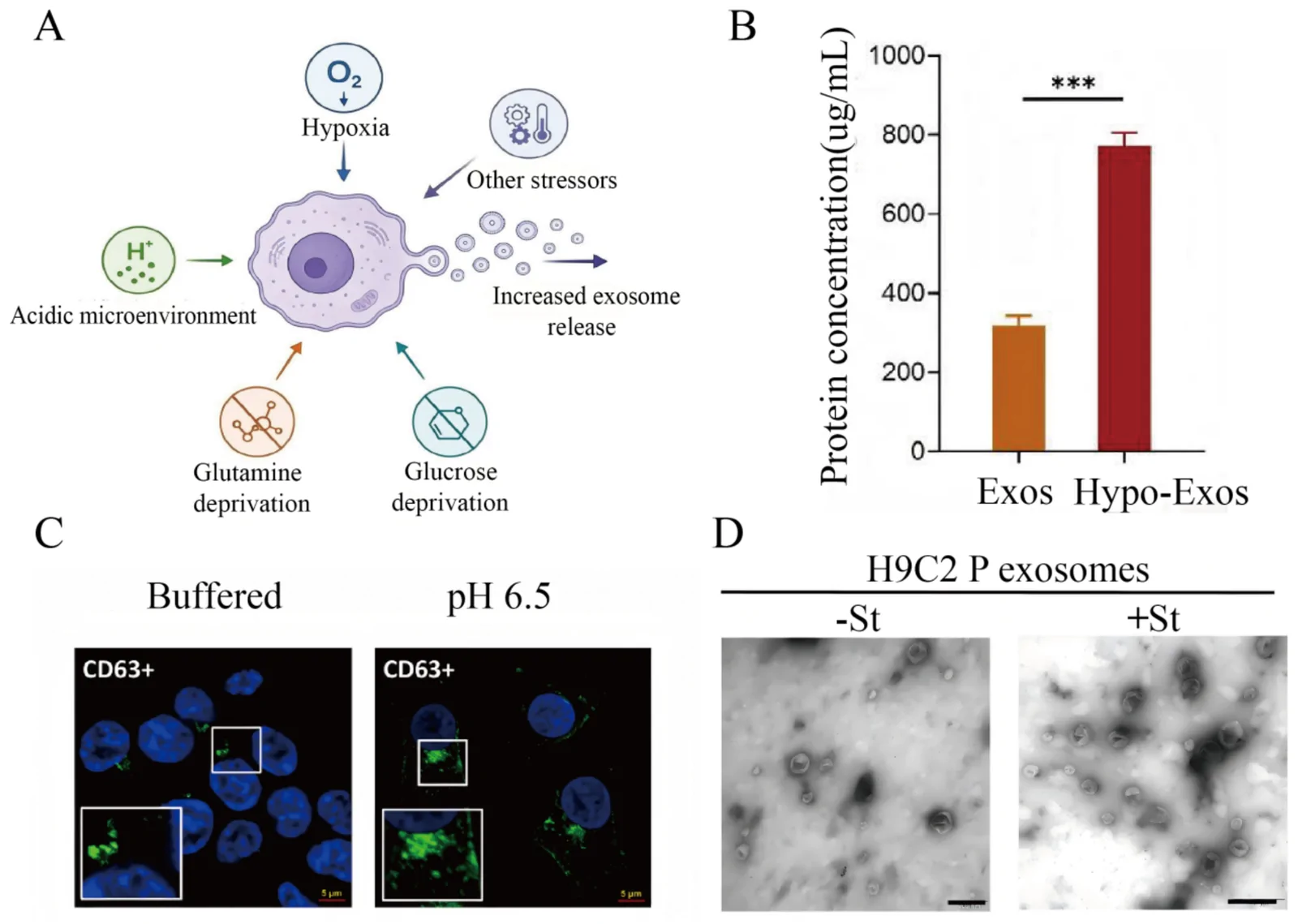
Environmental factors promote EVs release. (**A**) Schematic of EVs release types induced by extracellular microenvironment and nutrient stress. (**B**) Increased EVs yield under hypoxia. Data are presented as mean ± SD. *** *p* < 0.001 compared with the Exos group. Reproduced from Ref. [58], Copyright 2025, Wiley-VCH GmbH (**C**) Confocal microscopy of tumor cells cultured in buffered and pH 6.5 conditions. The cells, after being fixed in 3% paraformaledehyde, were labeled with CD63 monoclonal antibody (MEM-259), and Alexa Fluor 488 with a concentration of 1:25 for 2 h at room temperature; then, they were labeled with diamidino-2-phenylindole (DAPI) and observed with a confocal microscope. (**Left**) Tumor cells cultured in buffered conditions labeled with CD63. (**Right**) Tumor cells cultured in pH 6.5 conditions labeled with CD63. Reproduced from Ref. [59], Copyright 2018, MDPI. (**D**) Glucose starvation increases EVs secretion in H9C2 cells. Representative electron microscopy images of d P EVs collected from 90 mL of conditioned medium from H9C2 cells grown for 48 h under glucose-starved (+St) or glucose-replete (−St) conditions. Scale bars, 200 nm. Reproduced from Ref. [60], Copyright 2015, PLOS.

The extracellular microenvironment and nutrient stress represent two core dimensions that regulate EV release. Through intricately intertwined signaling networks, they collectively constitute the fundamental logic underlying the “secretory reprogramming” of tumor and stressed cells (Figure 7A). Numerous studies have demonstrated that physicochemical factors in the tumor microenvironment—such as hypoxia, oxidative stress, and acidic pH—as well as nutrient stresses like glucose or glutamine deprivation, can significantly promote the release of extracellular vesicles, particularly EVs, and profoundly remodel their molecular cargo and biological functions. Although the initial sensors of these stimuli differ—hypoxia acts through the HIF-1α transcriptional axis, oxidative stress through the ROS-TFEB signaling cascade, acidification through proton sensing and ion channels, and nutrient deprivation through the mTORC1-autophagy pathway—the core mechanisms by which they promote EV release converge on a common pathway: they all disrupt lysosomal homeostasis and the multivesicular body (MVB) degradation pathway, redirecting the fate of MVBs from “lysosomal degradation” toward “plasma membrane fusion and extracellular release,” thereby playing critical roles in tumor progression, angiogenesis, and tissue repair.

The hypoxic microenvironment serves as a critical biological switch driving the efficient release of extracellular vesicles, with the core mechanism relying on the transcriptional regulation by hypoxia-inducible factor HIF-1α. Studies have shown that HIF-1α can significantly increase EV production and bioactivity through two synergistic pathways: on one hand, it directly downregulates the expression of ATP6V1A, a subunit of the lysosomal proton pump, thereby impairing lysosomal degradation function and reducing the fusion of multivesicular bodies (MVBs) with lysosomes, which allows intraluminal vesicles to be secreted as EVs [61]; on the other hand, HIF-1α transcriptionally activates HRS, a key component of the ESCRT complex, which enhances endosomal sorting efficiency, further suppresses MVB degradation, and alters the packaging of exosomal cargo proteins [62]. The coordinated action of these two pathways not only substantially increases EV quantity but also enriches their load of pro-angiogenic and osteogenesis-related active molecules. Notably, hypoxia and microenvironmental acidification often reinforce each other—hypoxia-induced glycolysis produces large amounts of lactic acid, which exacerbates acidification, and acidification itself can also promote EV release, forming a positive feedback loop that continuously amplifies the HIF-1α-driven EV secretion effect. Based on this biological rationale, Luo et al. in a recent study utilized hypoxia-preconditioned human umbilical vein endothelial cell (HUVEC)-derived EVs (HExo) and confirmed that under hypoxic conditions, exosomal protein concentration, particle number, and cellular uptake efficiency were significantly higher than those under normoxic conditions (Figure 7B), and their pro-angiogenic capacity was enhanced in a dose-dependent manner. The team further encapsulated HExo into PEGDA/GelMA dual-network hydrogel microspheres via microfluidic technology to achieve sustained release for up to 18 days, and combined these with 3D-printed biomimetic trabecular titanium scaffolds (BTPS), successfully constructing a dual-biomimetic bone repair system that integrates structural mimicry with spatiotemporal control of bioactive molecule release [58]. This strategy not only validates the functional superiority of hypoxia-derived EVs but also provides a paradigm for transforming endogenous hypoxia signals into controllable therapeutic vehicles, fully demonstrating the organic extension from fundamental mechanisms to translational medicine.

Within the framework of metabolic stress, oxidative stress serves as another key hub linking metabolic disturbances to vesicle trafficking. Duan et al. [63] revealed a complete signaling axis in hepatocellular carcinoma: reactive oxygen species (ROS) stimulate the nuclear translocation of transcription factor EB (TFEB), which in turn upregulates the expression of β-hexosaminidase B (HEXB) and its antisense lncRNA HEXB-AS. HEXB-AS binds to the HEXB protein to form a protein/RNA complex, enhancing the protein stability of HEXB. The stabilized HEXB further interacts with lysosomal-associated membrane glycoprotein 1 (LAMP1), disrupting the fusion of lysosomes with MVBs, allowing MVBs that would otherwise be degraded by lysosomes to escape and be released as EVs. The ingenuity of this mechanism lies in the fact that oxidative stress, through TFEB—the “master regulator” of lysosomal biogenesis—paradoxically triggers a “selective inactivation” of lysosomal function: while promoting lysosomal gene transcription, it specifically blocks the degradation capacity of lysosomes toward MVBs through the interference of HEXB with LAMP1, thereby achieving a net increase in EV release.

Microenvironmental acidification is another important dimension. The aberrant fermentative metabolism of tumor cells leads to substantial accumulation of lactate and H^+^, creating the hallmark of extracellular acidification. The pioneering work of Parolini et al. first revealed that low pH is an important marker of tumor malignancy and can significantly affect the release and uptake of tumor-derived EVs [64]. Subsequently, Logozzi et al. [59] systematically demonstrated in five human tumor cell lines from different tissue origins that lowering the culture pH from the physiological 7.4 to the tumor-typical 6.5 significantly increased EV release in all cell lines (Figure 7C); conversely, buffering the acidic environment significantly reduced EV release in all tested cancer cells, providing the first evidence that tumor acidity and EVs levels represent a common cancer phenotype across tissue types. Mechanistically, acidification not only increases EVs quantity but also alters their physical properties—EVs released under acidic conditions exhibit higher membrane rigidity and increased sphingomyelin/ganglioside GM3 content, which enhances their fusion efficiency with recipient cells. Boussadia et al. [65] further discovered that EVs released by melanoma cells in an acidic microenvironment are enriched with proteins closely associated with poor prognosis, such as HRAS, HSP90B1, and NRAS, and are capable of conferring migratory and invasive abilities onto melanoma cells that were previously “naive” to pH.

Unlike the physicochemical factors mentioned above, nutrient stresses such as glutamine and glucose deprivation represent a parallel regulatory dimension. Fan et al. [66] uncovered a more refined regulatory layer: glutamine deprivation or Akt/mTORC1 signaling inhibition induces cancer cells to shift from “classical” CD63-positive late endosome-derived EVs to “stress-induced” Rab11a-positive recycling endosome-derived EVs. These Rab11a-positive EVs carry a unique molecular cargo, including the EGFR ligand amphiregulin (AREG), which can promote the proliferation of recipient tumor cells through the ERK-MAPK signaling pathway. Zou et al. [67] further demonstrated at the regulatory level that EVs release is negatively regulated by mTORC1—sustained activation of mTORC1 inhibits EVs release, leading to the intracellular accumulation of CD63-positive EVs precursors; whereas inhibition of mTORC1 by rapamycin or nutrient/growth factor deprivation stimulates EVs release. Glucose deprivation has also been proven to effectively drive EVs release. Garcia et al. [60] found that cardiomyocytes significantly increase EVs secretion under glucose starvation conditions, and that the miRNA and protein cargo of these EVs undergoes systematic remodeling in response to glucose availability—after glucose deprivation, the number of exosomal miRNA species increased from 13 to 30, among which pro-angiogenic miRNAs such as miR-17, miR-19a, and miR-126 were significantly upregulated, capable of inducing the transcriptional activation of multiple pro-angiogenic genes in endothelial cells and promoting endothelial cell proliferation and tube formation (Figure 7D).

It is noteworthy that the EVs-releasing effects induced by these different forms of stimuli do not operate in isolation, but are coupled through common signaling hubs. The gene network upregulated by HIF-1α under hypoxia also encompasses a large number of lysosome-related genes; there is interplay between oxidative stress and acidification—TFEB itself is an acid-sensitive transcription factor; and the inhibition of mTORC1 by glutamine deprivation has a bidirectional regulatory relationship with oxidative stress, where mTORC1 activity directly affects ROS levels, and ROS in turn regulate mTORC1. The crosstalk among these signaling pathways constitutes a complex regulatory network, enabling diverse microenvironmental and nutrient stress signals to converge on the common execution node of the lysosome-MVB axis.

In summary, diverse microenvironmental and metabolic stresses—including hypoxia, oxidative stress, acidification, glutamine deprivation, and glucose starvation—all converge on the lysosome–MVB axis to redirect MVB fate from degradation to secretion. Although each stress engages distinct upstream sensors (e.g., HIF-1α, ROS–TFEB, proton sensing, or mTORC1), their downstream execution uniformly impairs lysosomal function or MVB–lysosome fusion, thereby boosting EV release. This section thus underscores the central role of lysosomal dysfunction and MVB rerouting in stress-induced EV secretion, while the broader mechanistic convergence and translational implications are summarized in the concluding section.

In summary, although biochemical intervention strategies—including genetic engineering, small-molecule compounds, bioactive macromolecules, and microenvironmental modulation—differ in their targets and pathways, they all converge on the core regulation of multivesicular body (MVB) biogenesis, trafficking, and the balance of lysosomal degradation, thereby significantly promoting extracellular vesicle (EV) release and often endowing the EVs with specific functional modifications. To facilitate a direct cross-comparison of the mechanisms and enhancement effects of these strategies, we have summarized the representative studies in Table 2.

## 4. Conclusions

In summary, the past decade has witnessed remarkable progress in strategies to enhance EV production, evolving from empirical observations to mechanism-driven engineering approaches. This review systematically categorizes these strategies into physical interventions—encompassing mechanical loading, electrical stimulation, acoustic stimulation, light stimulation, and temperature modulation—and biochemical approaches, including genetic engineering, small-molecule compounds, bioactive macromolecules, and microenvironmental/nutrient stress modulation. Despite the apparent diversity of these inducers, a unifying mechanistic framework emerges: virtually all effective stimuli converge on common hub pathways that ultimately redirect the fate of multivesicular bodies (MVBs) from lysosomal degradation toward plasma membrane fusion and extracellular release. Physical signals operate through mechanotransduction and cytoskeletal remodeling, calcium influx, or membrane perturbation, while biochemical cues act via transcriptional reprogramming, lysosomal acidification interference, or receptor-mediated signaling cascades. Notably, the lysosome–MVB axis, the cytoskeleton, and calcium signaling represent three interconnected core checkpoints through which most enhancement strategies exert their effects.

An important insight from this comprehensive analysis is that yield enhancement does not necessarily compromise quality. Multiple studies have demonstrated that EVs derived from stimulated donor cells retain their characteristic size, morphology, marker expression, and biological functions, and in many cases even acquire enhanced therapeutic cargo—such as pro-angiogenic miRNAs, osteogenic mRNAs, or immunomodulatory proteins—depending on the stimulation modality. This opens exciting opportunities for “dual-purpose” engineering: simultaneously boosting quantity and tailoring functionality.

However, critical challenges remain. First, the heterogeneity of response across cell types and stimulation parameters calls for systematic, standardized protocols to ensure reproducibility and scalability. Second, the translation of these strategies from laboratory-scale to industrial-scale production requires integration with bioreactor systems, quality-by-design principles, and robust analytical methods for product characterization. Third, the potential off-target effects of certain stimuli—particularly prolonged chemical or thermal stress—on the molecular fingerprint of EVs necessitate rigorous safety and efficacy evaluations prior to clinical application. Fourth, the comparative effectiveness and cost–benefit ratios of different strategies remain largely unexplored, hindering informed decision-making for specific therapeutic contexts.

## 5. Future Perspectives

Looking forward, we envision that future breakthroughs will arise from the rational combination of complementary strategies—for example, integrating genetic engineering with three-dimensional culture systems, or coupling acoustic stimulation with biomaterial scaffolds—to achieve synergistic enhancement while minimizing undesirable side effects. The convergence of advanced manufacturing, artificial intelligence-driven process optimization, and deeper mechanistic understanding of EVs biogenesis will undoubtedly accelerate the clinical translation of EVs-based therapies. Ultimately, the goal is not merely to produce more EVs, but to produce better EVs—with precisely controlled potency, targeting, and safety profiles—ushering in a new era of cell-free regenerative medicine and precision therapeutics.

Looking further ahead, the integration of yield-enhancing strategies with complementary functional engineering approaches may unlock additional translational opportunities. For instance, Ding et al. demonstrated that non-discriminating iron oxide hydroxide nanomask shielding of CD47 on tumor-derived EVs enhanced phagocytosis and STING activation without altering EV yield, while Yu et al. developed ACTIVITY, an electrochemical cascade-amplification platform for profiling EV metabolic activity with ultrasensitive detection capabilities [68,69]. Although these studies do not directly increase EV production, they highlight complementary strategies [70,71] that, when combined with the secretion-enhancing approaches reviewed here, could enable integrated EV manufacturing workflows with real-time quality control and functional readouts. Such convergence of production engineering, surface functionalization, and smart detection will be instrumental in propelling EV-based therapies and diagnostics toward clinical reality.

## Figures and Tables

**Table 1:** Physical approaches for enhanced EV secretion.

Stimulation Type	Cell Type	Simplified Mechanism	Quantitative Method	Normalization	Method of EV Isolation	Fold Range	References
Mechanical loading	RAW264.7/HeLa	Mechanical force → cytoskeletal remodeling → MVB anchoring and fusion → EV release ↑.	NTA/BCA/FACS	Noralized by cell count	Differential ultracentrifugation/Kit	2–20-fold	Huo et al. [21]; Wan et al. [17]; Ji et al. [19]
Electrical stimulation	BV2 macrophages/MSCs	Electrical stimulation → Ca^2+^ influx ↑ → ILV enrichment and fusion in MVBs ↑ → EV release ↑	NTA/BCA	Normalized by cell count	Differential ultracentrifugation/Kit	8–24-fold	Fu et al. [29]; Peng et al. [30]; Zhang et al. [28]
Acoustic stimulation	ADSCs/U87-MG/HA/A549	Acoustic stimulation → membrane permeability ↑/Ca^2+^ influx → ESCRT pathway activation → MVB formation and fusion ↑ → EV release ↑	NTA/Western blot/BCA	Normalized by cell count	Ultrafiltration/Kit	Several-fold to ~10-fold	Ambattu et al. [35]; Zheng et al. [34]; Deng et al. [36]
Light stimulation	DC/MSCs	Photostimulation → intracellular signaling activation → inhibition of MVB-lysosome fusion/enhancement of MVB formation ↑ → EV release ↑	NTA/BCA/NanoView single-EV microarray	Normalized by cell count	Differential ultracentrifugation/Kit	2.7–13.3-fold	Ruan et al. [38]; Wu et al. [39]
Temperature modulation	HEK293F/MCF-7/BMMC	Hyperthermia/heat treatment → activation of heat shock proteins (HSPs), Rab GTPases, and ESCRT pathways → enhancement of MVB formation, fusion, and trafficking ↑ → EV release↑	NTA/BCA/TEM	Normalized by cell count	Differential ultracentrifugation/Kit	~6-fold	Keysberg et al. [42]; Xu et al. [40]; Liao et al. [41]

The upward arrows indicate upregulation, and the horizontal arrows indicate the next step.

**Table 2:** Biochemical approaches for enhanced EVs secretion.

Stimulation Type	Cell Type	Simplified Mechanism	Quantitative Method	Normalization	Method of EV Isolation	Fold Range	References
Genetic engineering	A549/H460/MPC5/hMSCs	CD147/KIBRA/BMP2 gene modulation → signaling activation/protein stabilization/mRNA loading → MVB formation, fusion, and secretion ↑ → EV release ↑.	NTA/Western blot/BCA	Normalized by cell count	Differential ultracentrifugation/Kit	2–3-fold	Yang et al. [45]; Song et al. [46]; Li et al. [42]
Small-molecule compounds	K562/B16.F10	Small-molecule drug → Ca^2+^ ↑/PERK-IRE1α activation → ↓ lysosomal acidification & ↓ MVB-lysosome fusion/↑ MVB-plasma membrane fusion →EV release ↑	NTA/BCA/Proteomics	Normalized by cell count	Differential ultracentrifugation/Kit	2–6-fold	Savina et al. [50]; Guo et al. [51]; Zhou et al. [52]
Bioactive macromolecules	HucMSCs/BMSCs	Cytokine preconditioning → STAT/ESCRT pathway activation/miRNA reprogramming → MVB formation and fusion ↑ → EV release ↑ + enhanced functionality	NTA/BCA/TEM	Normalized by cell count	Differential ultracentrifugation/Kit	1.5–5-fold	Chen et al. [53]; He et al. [54]; Pu et al. [56]
Microenvironmental modulation	LNCaP/SaOS2/H9C2	Environmental change → stress signaling activation/miRNA reprogramming/proteomic remodeling → MVB formation and fusion ↑ → EV release.↑	NTA/BCA/TEM	Normalized by cell count	Differential ultracentrifugation/Kit	~6–102-fold	Wang et al. [57]; Logozzi et al. [64]

Upward arrows denote upregulation, whereas horizontal arrows denote the next step.

## Data Availability

No new data were created or analyzed in this study. Data sharing is not applicable to this article.

## Abbreviations

**Abbreviation**	**Full term**
2D	two-dimensional
3D	three-dimensional
ALIX	ALG-2-interacting protein X (apoptosis-linked gene 2-interacting protein X)
ATF4	activating transcription factor 4
BMP2	bone morphogenetic protein 2
EGF	epidermal growth factor
ERK	extracellular signal-regulated kinase
ESCRT	endosomal sorting complexes required for transport
EVs	extracellular vesicles
FUS	focused ultrasound
GelMA	gelatin methacryloyl
HGF	hepatocyte growth factor
HIF-1α	hypoxia-inducible factor 1-alpha
IL-1β	interleukin-1 beta
ILVs	intraluminal vesicles
IRE1α	inositol-requiring enzyme 1 alpha
LAMP1	lysosomal-associated membrane glycoprotein 1
MAPK	mitogen-activated protein kinase
mTORC1	mechanistic target of rapamycin complex 1
MVBs	multivesicular bodies
